# Osteoarthritis of the Wrist STT Joint and Radiocarpal Joint

**DOI:** 10.1155/2012/242159

**Published:** 2012-08-26

**Authors:** Ronit Wollstein, Julio Clavijo, Louis A. Gilula

**Affiliations:** ^1^Division of Plastic and Reconstructive Surgery, Department of Orthopaedic Surgery and Department of Surgery, University of Pittsburgh Medical Center, 3550 Terrace St., Pittsburgh, PA 15261, USA; ^2^Mallinckrodt Institute of Radiology, Barnes-Jewish Hospital, Washington University School of Medicine, 510 South Kingshighway, St. Louis, MO 63110, USA

## Abstract

Our understanding of wrist osteoarthritis (OA) lags behind that of other joints, possibly due to the complexity of wrist biomechanics and the importance of ligamentous forces in the function of the wrist. Scaphotrapeziotrapezoidal (STT) OA is common, but its role in wrist clinical pathology and biomechanics is unclear. We identified the prevalence of radiographic STT joint OA in our hand clinic population and defined the relationship between STT and radiocarpal OA in wrist radiographs. One hundred consecutive wrist clinical and radiographic exams were retrospectively reviewed. Radiographs were evaluated for the presence and stage of OA. The mean age was 61.3 (±14.5) years. The radiographic occurrence of STT joint OA was 59% and of radiocarpal (RC) OA was 29%. Radiographic STT and RC joint OA were inversely related. Tenderness over the STT joint in physical exam was not associated with OA in the STT or other joints. STT OA in our series was not related to wrist pain. These findings support the discrepancy between radiographic and cadaver findings and clinically significant OA in this joint. The inverse relationship between STT and RC OA, as seen in scapholunate advanced collapse (SLAC) wrist, requires further biomechanical study.

## 1. Introduction

The role that the scaphotrapeziotrapezoidal (STT) joint plays in the biomechanics of the wrist joint and thumb is still uncertain. Therefore, the behaviour of this joint, when involved in osteoarthritis (OA), is understood even less. Furthermore, the magnitude of clinically significant (tender, painful) STT OA has not been established.

OA in the STT joint is the second most common site of radiographic OA in the wrist, reported in 15–29% of wrist radiographs, yet a higher occurrence (up to 83.3%) has been documented in cadaver studies [1–3]. Since the prevalence of clinical STT joint arthritis is estimated in some studies to be about 11%; it is possible that radiographs underestimate the actual (cadaveric) occurrence of STT joint OA, and that furthermore, most cases of STT OA are not clinically significant and therefore remain undiagnosed [4]. Based on clinical observation, we hypothesize that the true incidence of STT joint OA is more common than the 15% described radiologically in the literature, and that radiographic OA in this joint is inversely related to the occurrence of radiographic OA in the radioscaphoid (RS) joint [3]. The specific aims of this study were to identify the prevalence of radiographic STT joint OA in the population seen in our hand clinic and to define the relationship between OA in the STT and the radiocarpal joints on the wrist radiographs taken in our hand clinic. Secondary goals included evaluating the relationships between the different radiographic findings (STT OA, radiocarpal OA, thumb CMC joint OA, and measurement of the scapholunate gap) and between tenderness on physical examination and radiographic findings.

## 2. Methods

One hundred standard wrist radiographic exams obtained consecutively in our hand clinic, and the patient charts were retrospectively reviewed. Posteroanterior (PA), lateral, and oblique views of the wrist were included. Radiographic exams were excluded if they were of insufficient quality for evaluation or if the examination did not include all of these views. Charts with an incomplete wrist examination were excluded from the study. We randomly included only one of the wrists in patients with bilateral exams. 

Demographic information was obtained including: age, gender, and relevant medical history such as diabetes, rheumatoid arthritis, hypothyroidism, renal failure, smoking, and alcoholism. The rationale for the wrist radiographic examination, wrist side, relevant wrist operations, trauma history, occupation, and information regarding the physical examination of the wrist were collected as well. All physical examinations were performed by the same single surgeon board certified examiner in hand surgery. Institutional review board (IRB) approval (exempt approved) was obtained prior to study commencement.

The radiographs were reviewed by the first author in a blinded fashion (prior to data collection from the chart). They were evaluated for the presence and stage of STT joint and radiocarpal joint OA as well as thumb carpometacarpal joint OA, cystlike defects, and the presence of generalized osteoporosis. 

The radiographic OA in the STT joint was classified using a classification system based on regular wrist radiographs [6]. This classification system does not include an evaluation of the trapeziotrapezoidal joint. It defines the stage by the highest stage regardless of joint (scaphotrapezial/scaphotrapezoidal) and regardless of the view. Stage 1 was defined as narrowing of the joint space compared with those of other intercarpal joints in the same radiograph, with or without periarticular sclerosis. Stage 2 was defined as narrowing of the joint space compared with other intercarpal joints in the same radiograph with or without periarticular sclerosis, and one or both: cystlike lucencies and/or osteophytes. Stage 3 was defined as complete narrowing of the joint.

Radiocarpal OA was defined according to the most prevalent progression pattern of wrist OA. The term for this pattern, scapholunate advanced collapse (SLAC), was coined by Watson and Ballet, and the stages in this study were categorized according to their classification [7] which was subsequently modified to include a stage 4 [8]. Stage 1 was defined as OA between the scaphoid and the radial styloid, stage 2 between the scaphoid and the scaphoid fossa, and stage 3 as OA between the lunate and the capitate. Stage 4 was added to include those radiographs with radiolunate arthritis or “panarthritis”. The reason OA of the wrist advances in this form remains unclear, though the inherent congruence of the joints (radiolunate more congruent than the radioscaphoid joint) and the common scapholunate ligament tear are important in the development of this pattern of wear [7]. Thumb CMC joint OA was defined by the method of Eaton and Glickel [9]. Stage 1 was defined as a normal radiological joint, stage 2 as joint narrowing with osteophytes smaller than 2 mm, stage 3 included joint narrowing with osteophytes greater than 2 mm in size, and stages 4 and 5 defined involvement of the scaphotrapezial, and the scaphotrapezoidal joints, respectively.

Scapholunate gap was considered enlarged if measured above 2 mm. The distance between the scaphoid and lunate was measured at midjoint of the scapholunate joint from the ulnar cortex of the scaphoid to the radial cortex of the lunate [10].

Parameters of history and physical examination included a history of radial-sided wrist pain and signs including swelling, tenderness over the radial styloid, anatomical snuff box, the STT joint, and the thumb CMC joint. Tenderness was evaluated by directly palpating the area involved, and each area was rated on a scale of 0–10 with 0 as no tenderness and 10 as maximal tenderness. The STT joint was palpated just distal and ulnar to the snuff box. Clinical information regarding the ulnar side of the wrist was recorded as well. Associations between radial wrist signs and symptoms and radiographic radial wrist pathology were analysed.

Statistical analysis included chi-square and *t*-test to assess correlations between the radiographic findings and between the radiographic findings and the clinical findings. The chi square-test was used for the evaluation of 2 nominal variables. Chi square test was used to describe the relationship between SLAC wrist and STT joint osteoarthritis. For this calculation, SLAC wrist OA was either stage 0 or stage 1–4, STT joint OA was either 0 or stage 1–3. The Wilcoxon Rank-Sum Test was used to correlate between appearance of radiocarpal and STT joint OA. A *P* value of ≤0.05 was considered significant. Unpaired *t*-test was used for the evaluation of continuous variables when comparing two groups.

## 3. Results

The mean age was 61.3 (±14.5) years with range of 24–89 years. The patient age had a normal distribution. The 95 percentile of the patients were within the average with a standard deviation of 14.5 years. Using the unpaired *t*-test the *P* value was 0.063 (−11.155; 0.431). The patient demographics are summarized in Table 1. Sixty-four patients were heavy laborers, and 36 worked in clerical jobs which included 5 homemakers. Sixty-two percent of the radiographs were taken because of wrist pain (Table 2).

The occurrence of radiographic STT joint OA, using the classification system [6], was 59%. Most of the identified STT joint arthritis was stage 1 (Table 3). The occurrence of radiographic RC OA was 29%. The majority of the identified RC arthritis was SLAC stage 1 (Table 4). Sixty-five patients had radiographic evidence of thumb CMC joint OA (Table 5).

No significant association was found between the occurrence of radiographic STT joint OA, or other radiographic abnormalities (RC OA, S-L gap), and patient characteristics: age (between 24 years and 89 years), gender, occupation, background disease, and smoking. Occupation (heavy labor) was significantly related to an increased S-L gap (*P* = 0.02). Male gender was significantly related to a radiographic RC OA (*P* = 0.05). Female gender was significantly related to thumb CMC joint OA (*P* = 0.02).

**Figure 1:**
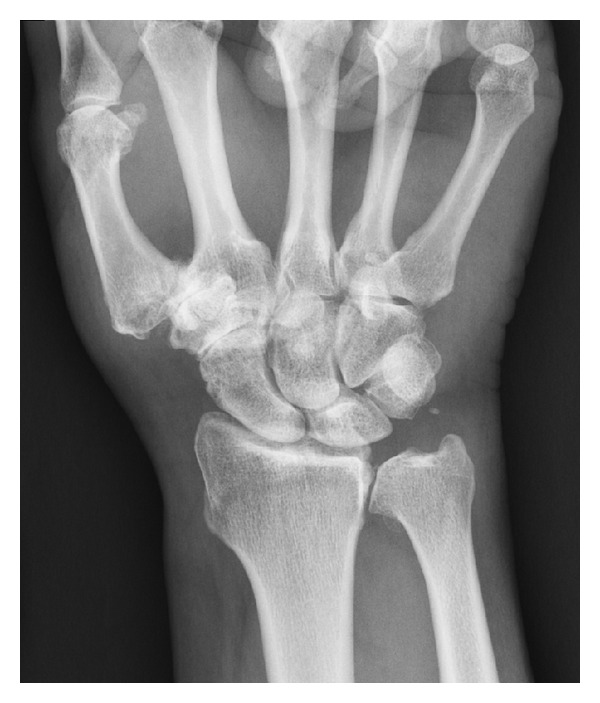
An example of STT joint arthritis stage 3 according to the classification system used. There is also thumb trapeziometacarpal arthritis and distal radioulnar joint arthritis. There is no evidence of radiocarpal arthritis.

STT joint OA and SLAC wrist on radiographs were found to be inversely related (*P* < 0.0001) (Figure 1).

There were 5 radiographic exams in which there was STT joint OA as well as RC OA. In three cases, the STT OA was stage 1. Two of these occurred with RC OA (SLAC) stage 2 and one with SLAC stage 3. There was one radiograph with stage 3 STT OA concurrent with stage 1 SLAC and one STT OA stage 2 concurrent with SLAC stage 1. The occurrence of radiographic capitolunate OA (not associated with RC OA) was 6% and was not found to be significantly related to STT joint OA (*P* = 0.63) or to the occurrence of an enlarged scapholunate gap (*P* = 0.25). STT joint OA was significantly related to thumb CMC joint OA (*P* = 0.05).

 When evaluating the parameters of physical examination and their relationship to the radiographic findings, tenderness to palpation over the STT joint was present in 18% of the patients. Only 10 patients with radiographic STT joint OA had tenderness over the joint with a mean of 0.59 (SD = 1.6) on a scale of 0–10 with a range of 0–8. Tenderness over the STT joint was not associated with OA in the STT joint, thumb CMC joint, or a SLAC wrist. The physical findings and their relationship to the radiographic findings are described in Table 6.

## 4. Conclusions

We found a 59% occurrence of STT joint OA. This is higher than in previous radiographic descriptions, though these numbers are closer to the occurrence found in cadaver studies [1, 7, 11]. In the study of Brown et al., they found poor concurrence (39%) between radiographic OA and OA found in the same cadavers [12]. It is possible that the use of the classification system for STT joint OA [6] as used in this work is more sensitive than general radiographic evaluation of OA in this joint where only stages 2 and 3 STT joint arthritis may have been identified as osteoarthritis. This classification may therefore produce results that are possibly closer to the actual occurrence of OA in the STT joint. 

Our average age was lower than that of Bhatia et al. where the average cadaver age was 84 [1]. In the study of Brown et al., the average age was 56.9 which is lower than that of our population [12]. It is plausible that age affects the occurrence of STT joint OA and therefore we may have found a higher occurrence of radiographic STT OA because of our population age. We would expect all wrist OA to increase with age, and the highest reported osteoarthritis occurrence was 83.3% in the series of Bhatia et al. (cadaver age was 84 years) [1]. However, we did not find a significant relationship between age and the occurrence of STT joint arthritis in this study. Since our age group was relatively uniform, with the great majority of our patients older (mean of 61.3 (±14.5) years), we may not have had enough power for statistical significance. Also, since we included relatively few young patients, this result may be misleading. It is not clear what the role is for age in the development of OA. Since wrist osteoarthritis in general is very rare in younger patients and is usually related to trauma, multiple factors are most likely responsible for its development. Further study would be necessary to better define the relationship between wrist OA and age.

This study verified many of the accepted principles for OA in the wrist. Our prevalence of RC arthritis or SLAC wrist, associations between RC OA and male gender, thumb CMC joint OA and female gender, as well as a significant association between STT joint OA and thumb CMC joint OA are all in tandem with the literature, as is a significant association between tenderness over the thumb CMC joint and OA in that joint and tenderness over the snuff box and RC OA [5, 12].

There was a strong association between some of our clinical parameters (more specifically point tenderness) and radiographic OA in the tender joint; RC OA (SLAC) wrist and tenderness over the anatomical snuff box, thumb CMC joint tenderness, and OA in the CMC joint, as well as an association between radial sided wrist pain and radiocarpal OA. The finding of STT joint OA in our series however was not related to wrist pain in any area. When tenderness over this area was elicited, it was very minimal even in the presence of STT joint OA. Some patients were tender over the area of the STT joint without evidence of radiographic OA. It could be that pain is elicited before the radiographic signs are apparent, or that it is referred to the STT area from adjacent joints such as the thumb CMC joint. This finding supports the discrepancy between radiographic and cadaver findings of STT joint OA and clinically significant OA in this joint. 

Possible explanations for this discrepancy are primarily biomechanical. It is possible that the arthritic STT joint moves very little and therefore does not cause pain (as opposed to the thumb CMC joint for example, that has 360 degrees of motion and is therefore more often painful in the presence of OA). It is also possible however that the arthritic STT joint is actually less stable but that because the scaphoid “slides” beneath the dome of the trapezium and trapezoid; load is preferentially transferred through the capitate and lunate and again causes no pain in the STT joint area. Other possible explanations include lack of pain receptors in the ligaments of the STT joint. Further study to elucidate the inconsistency between clinical and radiographic and cadaveric STT joint OA is needed.

In this study, the existence of radiographic radioscaphoid osteoarthritis was inversely related to the existence of STT joint osteoarthritis. The significance of this finding is unclear, and a biomechanical study would be necessary to investigate the implications of this relationship on the development of wrist OA.

The fact that this was a retrospective review limits the observations to patients requiring a wrist radiographic examination. Since our radiographs and clinical examinations are in tandem with the descriptions in the literature, we can assume that the population seen in our hand clinic is similar to that in most hand clinics. This may not however give an indication of the occurrence of radiographic arthritis in the general population (not seeing a hand surgeon and therefore not obtaining wrist radiographs). 

In summary, our higher occurrence of STT joint OA may be due to a more inclusive definition of STT OA. However, the lack of association with the clinical presentation of STT OA may signify that most instances of STT OA are asymptomatic or very mildly symptomatic. The inverse relationship between radiographic STT OA and RC OA warrants further study.

## Figures and Tables

**Table 1:** Population demographics.

	*N* = %
Gender (male)	81
Occupation (laborer)	64
Dominant = painful hand	55
Background disease (diabetes, rheumatoid arthritis)	24
Smoking	33
Alcohol use	9

**Table 2:** Clinical reasons for radiographs; *N* = 100.

	*N* = %
Wrist pain ulnar (isolated-triangular fibrocartilage tears)	21
Wrist pain radial (isolated)	29
Wrist pain radial and ulnar	12
Thumb base pain	12
Ganglion	4
Trauma distal radius fractures, crush, and metacarpal fractures	13
Trauma soft tissue/laceration	6
Soft-tissue insect bite, infection	3

**Table 3:** The occurrence of the stages of STT joint arthritis according to the radiographic classification system.

	*N* = (*N* = 100)	% (*N* = 59)
Stage I	28	47.4
Stage II	16	27.1
Stage III	15	25.4

**Table 4:** Occurrence of radiocarpal (RC) or scapholunate advanced collapse (SLAC) wrist by stages.

	*N*=	%
Stage 1	13	45
Stage 2	9	31
Stage 3	5	17
Stage 4	2	6
No SLAC wrist	71	—

Stage 4 is defined as arthritis including the radiolunate joint [5]. *N* = 100. The percentages pertain to number of radiographs by stage out of the 29 radiographs that had RC arthritis.

**Table 5:** Occurrence of thumb carpometacarpal (CMC) joint osteoarthritis (OA) by stages.

	*N*=	%
Stage 1	22	34
Stage 2	13	20
Stage 3	14	22
Stage 4-5	16	25
No CMC joint OA	35	—

*N* = 100, the percentages pertain to number of radiographs by stage out of the 65 radiographs that had thumb CMC joint arthritis.

**Table 6:** Physical examination parameters and their relationship to the radiographic findings (*P* values).

	STT osteoarthritis	SLAC	CMC osteoarthritis
Radial wrist pain	0.2863 (1.137)	<0.003 (8.366)	0.464 (0.534)
Swelling	0.5264 (0.401)	0.176 (1.825)	0.980 (4.72)
Radial styloid tend	0.89 (−1.020; 0.888)	0.120 (−1.828; 0.253)	0.297 (−1.542; 0.476)
Snuff box tend	**0.008 (0.500; 3.257)**	**<0.001 (−5.537; −2.948)**	**0.0063 (−3.522; −0.596)**
STT joint tend	0.45 (−0.398; 0.878)	0.272 (−1.072; 0.306)	0.816 (−0.760; 0.600)
CMC joint tend	0.23 (−2.326; 0.580)	0.139 (−0.390; 2.748)	**<0.001 (1.959; 4.765)**

Significant relationships are highlighted in bold type; tend: tenderness; SLAC: scapholunate advanced collapse; STT: scaphotrapeziotrapezoidal; CMC: 1st carpometacarpal. Results expressed as *P* value (chi-square) for radial wrist pain and swelling. Tend: tenderness over that area of the wrist on physical examination and documented as a continuous variable (0–10). Results expressed as *P* value (95% lower; 95% upper).
